# Knowledge and attitudes about transcranial magnetic stimulation among psychiatrists in China

**DOI:** 10.1186/s12888-020-02817-4

**Published:** 2020-08-24

**Authors:** Jiahui Deng, Yimiao Gong, Xiao Lin, Yanping Bao, Hongqiang Sun, Lin Lu

**Affiliations:** 1 Peking University Sixth Hospital, Peking University Institute of Mental Health, NHC Key Laboratory of Mental Health (Peking University), National Clinical Research Center for Mental Disorders (Peking University Sixth Hospital), Chinese Academy of Medical Sciences Research Unit (No.2018RU006), Peking University, 51 Huayuanbei Road, Beijing, 100191 China; 2 Peking-Tsinghua Center for Life Sciences and PKU-IDG/McGovern Institute for Brain Research, Peking University, Beijing, China; 3 National Institute on Drug Dependence and Beijing Key Laboratory on Drug Dependence Research, Peking University, Beijing, China

**Keywords:** Repetitive transcranial magnetic stimulation, Psychiatrists, Knowledge, Attitude, Formal training

## Abstract

**Background:**

Repetitive transcranial magnetic stimulation (rTMS) is a noninvasive form of brain stimulation. It has been used in many mental health institutions to treat mental disorders worldwide. However, comprehensive knowledge about rTMS is not yet widespread among psychiatrists. The present study assessed psychiatrists’ knowledge and attitudes about rTMS in China and investigated related factors.

**Methods:**

A quantitative observational cross-sectional study was conducted using an online survey. The sample consisted of 522 psychiatrists. Multinomial logistic regression and multiple linear regression analyses were used to explore factors that contributed to psychiatrists’ knowledge about rTMS. We also ascertained psychiatrists’ attitudes about rTMS and provide recommendations for the more widespread use of rTMS.

**Results:**

The majority of respondents (86.4%) reported having access to rTMS at their institution. A total of 379 psychiatrists (72.6%) knew that rTMS was approved by the United States Food and Drug Administration for treatment-resistant depression. Univariate logistic regression indicated that psychiatrists who were older, had a senior professional title, worked more years, had an onsite clinical rTMS program in their hospital, and received formal training in theory and application (all *p* <  0.05) were more likely to know that rTMS was approved by the Food and Drug Administration for the treatment of depression. The percentages of respondents who knew most or all indications, the mechanism of action, parameter settings, adverse reactions were 51.9, 40.2, 27.4, and 41.4%. Linear regression showed that formal training in rTMS theory and practice were associated with higher knowledge scores (all *p* <  0.05). Most of the subjects had negative attitudes about using rTMS to treat mental disorders. When asked about their attitudes about continuing rTMS education, nearly all of the respondents indicated that they were willing to pursue continuing training in rTMS in the future.

**Conclusions:**

Many psychiatrists had an insufficient level of knowledge about rTMS and negative attitudes about rTMS. Psychiatrists who had formal rTMS training experience had higher levels of rTMS knowledge. rTMS training and relevant policy making should be strengthened.

## Background

Repetitive transcranial magnetic stimulation (rTMS) is a noninvasive form of brain stimulation. An electromagnetic coil is placed near the scalp, and repetitive pulses of electric current generate high magnetic fields and create an electric field within the brain. rTMS is an approved noninvasive neuromodulation technique that activates or inhibits cortical activity [1]. Numerous clinical trials have confirmed that rTMS effectively treats mental disorders, such as depression, obsessive-compulsive disorder, auditory hallucinations, and negative symptoms of schizophrenia [2–4]. Health authorities in many countries (e.g., United States, Canada, Australia, and Germany) have approved rTMS as a treatment for depressive disorders. The U.S. Food and Drug Administration (FDA) also approved the application of deep TMS for the treatment of obsessive-compulsive disorder. Evidence-based guidelines on the therapeutic use of rTMS have fostered the increasing recognition of rTMS as a treatment option [5]. A recent scientometric analysis found that among neurostimulation therapies, rTMS had the second-highest popularity index in psychiatry [6]. In China, rTMS has been used in hundreds of psychiatry departments to improve symptoms of mental disorders.

Psychiatrists’ and patients’ knowledge and attitudes about a physical therapy is correlate with therapeutic response [7, 8]. Patients’ misconceptions about a treatment modality are directly affected by their physicians’ knowledge about such treatments [9]. However, comprehensive knowledge about rTMS is not yet widespread among psychiatrists. A previous study asked respondents in three U.S. hospitals if they knew how to refer patients for rTMS, and two-thirds of the respondents reported that they did not know [10]. Psychiatrists were poorly informed about, and poorly trained in, rTMS and reported a desire to receive more training and information [11]. By the end of 2015, China had 2936 mental health institutions and 30,122 licensed psychiatrists [12]. Guidelines and expert consensus have indicated that rTMS is an effective treatment option, such as for depressive disorder and insomnia disorder. Unclear, however, are Chinese psychiatrists’ knowledge and attitudes about rTMS. The present study examined Chinese psychiatrists’ knowledge and attitudes about rTMS and analyzed the factors that influence such knowledge and attitudes.

## Methods

### Study design and setting

An anonymous quantitative observational cross-sectional study was performed using the WeChat-based survey program Questionnaire Star in October 2019 in China. We directed questionnaires to psychiatrists who worked in specialized psychiatric hospitals or in psychiatry departments of general hospitals and included all psychiatrists who agreed to participate in the self-administered online survey. Prior to enrollment, the participants were told that their consent to participate in the study would be assumed if they completed the online survey.

### Questionnaire

A self-designed questionnaire that investigated knowledge and attitudes about rTMS among psychiatrists was used in the present study because we are unaware of such a questionnaire that has been previously validated (Table S1). The duration of completing questionnaire was ~ 5 min. Four sections were included in the questionnaire: sociodemographic details, rTMS knowledge, attitudes about rTMS, and recommendations for rTMS development in the future. In the first section, we collected demographic information, including age, gender, years of education, educational background, professional title, attributes of the department and hospital, and whether the psychiatrists received training in rTMS theory and application. In the second section, we asked the participants whether they had learned about FDA indications for rTMS for treatment-resistant depression, and the respondents answered “yes” or “no.” We asked the participants about their rTMS knowledge, including indications, principles of treatment, parameter settings, adverse reactions, and contraindications, and the respondents answered “know nothing or a little,” “know some,” or “know most or all.” In the third section, we asked the respondents whether they would recommend rTMS for the treatment of mental disease either alone or combined with other treatments, and the respondents responded on a 5-point Likert scale (“strongly discourage,” “don’t recommend,” “neutral,” “recommend,” and “strongly recommend”). A multiple-choice question about the reasons why they chose “don’t recommend” or “strongly discourage” had six response options: “no effect or limited effect,” “slow onset,” “not covered by medical insurance,” “side effects,” “do not know how to develop a treatment plan,” and “other.” We also asked a multiple-choice question about recommendations for rTMS application, such as medical insurance, knowledge popularization, clinical practice, and other.

### Data analysis

Descriptive statistical data are presented as means, standard deviations, and percentages. Variables were included in the multivariate logistic regression analysis if they had values of *p* <  0.05 in the univariate analysis. Associations between factors and outcomes are presented as odds ratios (ORs) and 95% confidence intervals (CIs). We used a linear regression model to assess associations between demographic characteristics and comprehensive rTMS knowledge scores. Values of *p* <  0.05 were considered statistically significant. The data were analyzed using SPSS 24.0 software.

## Results

### Demographic characteristics

A total of 522 psychiatrists from 30 provinces and autonomous regions in China completing the questionnaire, of which 268 (51.3%) were male and 254 (48.7%) were female, with a mean age of 38.7 ± 8.5 years. Demographic characteristics of the psychiatrists are shown in Table 1. The proportions of specialty hospitals and psychiatric departments in general hospitals were 72.2 and 27.8%, respectively. A total of 230 psychiatrists (44.1%) received formal training in rTMS theory, and 149 psychiatrists (28.5%) applied rTMS in their clinical practice. A total of 451 psychiatrists (86.4%) reported having access to rTMS within their clinical departments, and 71 psychiatrists (13.6%) reported that they did not have access or were unsure about access.

**Table 1:** Demographic characteristics of the respondents

Demographic characteristic	No. (%) of Respondents
**Gender**	
Men	268 (51.3)
Women	254 (48.7)
**Age**	
21–30 years	66 (12.6)
31–40 years	236 (45.2)
41–50 years	147 (28.2)
> 50 years	73 (14.0)
**Professional title**	
Resident doctor	132 (25.3)
Attending physician	205 (39.3)
Associate chief physician	125 (23.9)
Chief physician	60 (11.5)
**Educational background**	
College	22 (4.2)
Bachelor’s degree	342 (65.5)
Master’s degree	113 (21.6)
Doctoral degree	45 (8.6)
**Years of work**	
1–10 years	214 (41.0)
11–20 years	168 (32.2)
> 20 years	140 (26.8)
**Type of hospital**	
Public hospital level I	44 (9.4)
Public hospital level II	149 (28.5)
Public hospital level III	329 (63.0)
**Psychiatric affiliation**	
General hospital	145 (27.8)
Specialized hospital	377 (22.2)
**Onsite clinical rTMS program**	
No	71 (13.6)
Yes	451 (86.4)
**Trained in rTMS theory**	
No	292 (55.9)
Yes	230 (44.1)
**Trained in rTMS manipulation**	
No	373 (71.5)
Yes	149 (28.5)
**Number of provinces and cities**	30

### Knowledge about rTMS

As shown in Table 2, the psychiatrists were asked whether they knew about the FDA’s approval of rTMS for treatment-resistant depression; 379 psychiatrists (72.6%) answered “yes,” and 143 (27.4%) answered “no.” We then performed binary logistic regression analysis to identify sociodemographic characteristics and relevant factors that were associated with knowledge about rTMS. In the univariate logistic regression analysis, several factors were independently associated with knowledge about rTMS, including age, having a senior professional title, working more years, having an onsite clinical rTMS program in their hospital, receiving formal theory education, and receiving professional training (Table 2).

**Table 2:** Univariate and multivariate logistic regression analysis of factors that were associated with knowledge about U.S. FDA approval of rTMS for treatment-resistant depression

Variable	Yes (***n*** = 379)	No (***n*** = 143)	Unadjusted OR (95% CI)	Adjusted OR (95% CI)
**Gender**				
Men	197 (0.74)	71 (0.26)	1	
Women	182 (0.72)	72 (0.28)	0.91 (0.62–1.34)	
**Age**				
21–30 years	45 (0.68)	21 (0.32)	1	
31–40 years	163 (0.69)	73 (0.31)	1.04 (0.58–1.87)	
41–50 years	109 (0.74)	38 (0.26)	1.34 (0.71–2.53)	
> 50 years	62 (0.85)	11 (0.15)	2.63 (1.15–6.00)*	
**Professional title**				
Resident doctor	89 (0.67)	43 (0.33)	1	
Attending physician	144 (0.70)	61 (0.30)	1.14 (0.72–1.83)	
Associate chief physician	94 (0.75)	31 (0.25)	1.45 (0.85–2.53)	
Chief physician	52 (0.87)	8 (0.13)	3.14 (1.37–7.19)**	
**Educational background**				
College	16 (0.73)	6 (0.27)	1	
Bachelor’s degree	249 (0.73)	93 (0.27)	1.00 (0.38–2.64)	
Master’s degree	81 (0.72)	32 (0.28)	0.95 (0.34–2.64)	
Doctoral degree	33 (0.73)	12 (0.27)	1.03 (0.33–3.25)	
**Years of work**				
1–10 years	143 (0.67)	71 (0.33)	1	1
11–20 years	121 (0.72)	47 (0.28)	1.28 (0.82–1.98)	1.18 (0.75–1.86)
> 20 years	115 (0.82)	25 (0.18)	2.28 (1.36–3.83)**	2.09 (1.23–3.56)**
**Type of hospital**				
Public hospital level I	31 (0.70)	13 (0.30)	1	
Public hospital level II	110 (0.74)	39 (0.26)	1.18 (0.56–2.49)	
Public hospital level III	238 (0.72)	91 (0.28)	1.10 (0.55–2.19)	
**Psychiatric affiliation**				
General hospital	102 (0.70)	43 (0.30)	1	
Specialized hospital	277 (0.73)	100 (0.27)	1.17 (0.77–1.78)	
**Onsite clinical rTMS program**				
No	41 (0.58)	30 (0.42)	1	
Yes	338 (0.75)	113 (0.25)	2.19 (1.31–3.67)**	
**Trained in rTMS theory**				
No	182 (0.62)	110 (0.38)	1	1
Yes	197 (0.86)	33 (0.14)	3.61 (2.33–5.59)***	3.50 (2.25–5.43)***
**Trained in rTMS manipulation**				
No	251 (0.67)	122 (0.33)	1	
Yes	128 (0.86)	21 (0.14)	2.96 (1.78–4.93)***	

*OR* Odds ratio, *CI* Confidence interval. *p < 0.05, **p < 0.01, ***p < 0.001

Multivariate logistic regression analysis showed that working for more than 20 years and receiving formal training in rTMS theory facilitated knowledge about the FDA’s approval of rTMS for treatment-refractory depression. Compared with respondents who were employed for 1–10 years, respondents who were employed for > 20 years were more likely to know about FDA approval (OR = 2.09, 95% CI = 1.23–3.59, *p* < 0.01). Receiving formal training in rTMS theory was associated with more knowledge about rTMS (OR = 3.50, 95% CI = 2.25–5.43).

A total of 51.9% of the respondents knew most or all indications for rTMS. Less than 50% of the respondents knew most or all principles of rTMS, parameter settings, adverse reactions, and contraindications (40.2, 27.4, and 41.4%, respectively; Table 3). We then performed multiple linear regression analysis to investigate the effects of age, gender, years of education, educational background, professional title, attributes of departments and hospitals, and receiving training in rTMS theory and application on psychiatrists’ knowledge about rTMS. In the model of comprehensive knowledge about rTMS, three variables (onsite clinical rTMS program in the hospital, having received training in rTMS theory, and having received training in rTMS application) were significant (all *p* < 0.001; Table 4), which explained 44.5% of the variance of knowledge about rTMS (adjusted *R*^*2*^ = 0.445, *p* < 0.001). These results indicate that theoretical training in rTMS was vital for the psychiatrists’ knowledge.

**Table 3:** Knowledge about rTMS among psychiatrists

Item	No. (%) of Respondents
**rTMS indications**	
Not knowing or knowing a little	146 (28.0)
Know part of	105 (20.1)
Know most or all	271 (51.9)
**rTMS principles**	
Not knowing or knowing a little	180 (34.5)
Know part of	132 (25.3)
Know most or all	210 (40.2)
**rTMS parameter settings**	
Not knowing or knowing a little	265 (50.8)
Know part of	114 (21.8)
Know most or all	143 (27.4)
**Adverse reactions and contraindications of rTMS**	
Not knowing or knowing a little	210 (40.2)
Know part of	96 (18.4)
Know most or all	216 (41.4)

**Table 4:** Multiple linear regression analysis of factors that influenced knowledge about rTMS

Variable	Unstandardized β	Standardized β	***t***	***p***
Gender				
Age	−0.18	−0.3	−0.89	0.37
Professional title	−0.03	− 0.01	− 0.13	0.89
Educational background	0.11	0.02	0.67	0.50
Year of work	−0.03	− 0.01	− 0.14	0.89
Type of hospital	0.29	0.09	1.68	0.09
Psychiatric affiliation	0.33	0.09	1.62	0.11
Onsite clinical rTMS program	1.24	1.14	3.92	< 0.001
Training in rTMS theory	2.51	0.41	9.68	< 0.001
Training in rTMS manipulation	1.65	0.24	5.73	< 0.001

### Attitudes about rTMS

Table 5 shows the psychiatrists’ attitudes about rTMS. We first asked whether the psychiatrists would recommend rTMS alone for patients with refractory mental disorders. Only 27 of the 522 respondents answered that they would strongly recommend this approach. This low likelihood had several reasons. First, the psychiatrists reported that rTMS has no effect or limited effect on mental disease (51.1%). Second, a slow onset of rTMS efficacy may delay treatment (51.1%). Third, the cost of rTMS is not covered by medical insurance in most parts of the country, which would place a financial burden on patients (29.8%). Fourth, some of the psychiatrists did not know how to design a treatment plan because of their lack of knowledge about rTMS (27.7%). The psychiatrists were then asked whether they would recommend rTMS as a combination therapy with other interventions to treat mental disorders. Only 53 of the 522 respondents (10.2%) answered that they would strongly recommend rTMS as an adjunct therapy to treat refractory mental disorders. The following reasons were given for not recommending rTMS. First, the psychiatrists reported that rTMS has no effect or limited effect on mental disease (66.7%). Second, a slow onset of TMS efficacy may delay treatment (50.0%). Third, some of the psychiatrists did not know how to design a treatment plan because of their lack of knowledge about rTMS (33.3%). Fourth, the cost of rTMS is not covered by medical insurance in most parts of the country, which would place a financial burden on patients (16.7%). The psychiatrists were then asked about their attitudes about continuing rTMS education, and nearly 100.0% of the respondents had a positive attitude. A total of 294 respondents (56.0%) reported that they would pursue continuing education training in rTMS certainly in the future (Table 5).

**Table 5:** Approval of rTMS application and continuing education

Item	No. (%) of Respondents
**rTMS alone for treatment of refractory mental disorders**	
Strongly discourage	7 (1.3)
Don’t recommend	40 (7.7)
Neutral	353 (67.6)
Recommend	95 (18.2)
Strongly recommend	27 (5.2)
**rTMS combined with other treatments for refractory mental disorders**	
Strongly discourage	0 (0.0)
Don’t recommend	6 (1.1)
Neutral	279 (53.4)
Recommend	184 (35.2)
Strongly recommend	53 (10.2)
**Psychiatrists need to know about TMS**	
Yes	518 (99.2)
No	4 (0.8)
**Participate in TMS training within hospital**	
Certainly not	1 (0.2)
Probably not	7 (1.3)
Probably	220 (42.1)
Certainly	294 (56.3)

### Recommendations for rTMS

To accelerate the clinical application of rTMS, we also asked the psychiatrists for their recommendations about rTMS. The results are shown in Table 6. A total of 86.4% of the respondents reported an urgent need to expand the scope of medical insurance reimbursement to include the cost of rTMS. A total of 371 respondents (71.1%) indicated the need to enhance the intensity of scientific research and optimize treatment plans for rTMS. A total of 77.0% of the respondents reported that formal training in rTMS theory and practice is also needed among psychiatrists to achieve standardized use. A total of 372 of the 522 respondents (71.3%) suggested formulating treatment specifications for rTMS. A total of 72.0% of the respondents recommended that rTMS should be popularized among patients.

**Table 6:** Recommendations for use of rTMS in the future

Recommendation	No. (%) of Respondents
Medical insurance coverage of rTMS	451 (86.4)
Enhance scientific research and optimize treatment plans	371 (71.1)
Formal training in rTMS theory and application among psychiatrists	402 (77.0)
Popularize rTMS among patients	376 (72.0)
Formulate treatment specifications for rTMS	372 (71.3)

## Discussion

The present study examined psychiatrists’ attitudes and knowledge about the application of rTMS in China. Our purpose was to evaluate Chinese psychiatrists’ knowledge about rTMS and provide recommendations for its future application as an effective intervention for refractory mental disorders in China. Although rTMS was reported to be currently used in most psychiatric departments and hospitals, approximately one-fourth of the psychiatrists were unaware of the FDA’s approval of rTMS for treatment-resistant depression, thus indicating a lack of knowledge about rTMS among Chinese psychiatrists. Additionally, most of the psychiatrists had only partial knowledge about rTMS, such as indications and mechanisms. The psychiatrists’ knowledge about rTMS was related to a variety of factors, including years of work, having rTMS onsite, and receiving rTMS training. Only one-tenth of the respondents strongly recommended TMS as an adjunct therapy for the treatment of refractory mental disorders.

Access to rTMS was common among the psychiatrists. Thus, one interpretation of the above finding is that psychiatrists may think that rTMS is less effective than electroconvulsive therapy (ECT). The latter is still the most popular neurostimulation treatment, with a high popularity index [6]. Frequent treatment with rTMS (20 sessions per month, 5 days per week) can cause great inconvenience for those who cannot come out from the workplace [13] and high financial burden for patients [14]. Furthermore, rTMS cannot be administered on a daily basis over the long term, and it lacks rapid antidepressant effects [15]. As a result, some psychiatrists prefer augmenting rTMS with different antidepressants or novel antidepressants with multimodal pharmacodynamic mechanisms of action [16, 17]. These findings revealed general limitations in psychiatrists’ knowledge and attitudes about rTMS as an emerging treatment option for treatment-resistant depression and other severe mental diseases. The responders recommended broader coverage of rTMS by medical insurers.

The present study demonstrated that formal training improved the psychiatrists’ perceived knowledge about rTMS. As expected, access to education affected psychiatrists’ knowledge about rTMS. This result is consistent with a previous study that found that psychiatrists with formal training in neuromodulation were significantly more likely to know and understand FDA indications for rTMS in treatment-resistant depression [10]. Because of their lack of knowledge about rTMS, many psychiatrists were willing to attend rTMS training in the future. These observations highlighted the need for further rTMS education and training among psychiatrists and graduate students in psychiatry. Structured neuromodulation training is also suggested within the field of psychiatry, and subspecialized training should be an area of expertise that requires subspecialty education and supervision [18]. Our recommendations support a defined educational approach for the standardized training of residents and practicing physicians. Despite an increasing number of studies on rTMS and increasing use in mental health institutions, rTMS is not consistently a requirement of residency education. In the future, psychiatry trainees should receive education on the neurobiology of mental disorders and basic training in neuromodulation techniques. Such training will improve the understanding of brain stimulation interventions and increase creative thinking about treatment options.

Our study highlights several future needs. First, the scope of medical insurance reimbursement should be expanded on the national scale. Second, rTMS is currently used in hundreds of hospitals, but unclear is whether all psychiatrists recognize its treatments benefits. Professional rTMS training also needs to be implemented in psychiatry departments with appropriate certification. Furthermore, the popularization of rTMS knowledge among patients is also indispensable, which will contribute to promotion of the clinical application of rTMS in China.

The present study has several limitations. First, it was a cross-sectional survey. Similar to previous studies [10, 19], we did not apply a standardized, interviewer-rated, and formal validated questionnaire. We asked psychiatrists to subjectively assess their knowledge about rTMS, which may not accurately reflect their true situation. Future surveys should ask respondents specific questions about rTMS using response options that are either correct or incorrect, with an additional response option of “I do not know.” Such a structured questionnaire would allow the evaluation of psychiatrists’ knowledge by calculating the total score. Second, our list of factors that may influence knowledge about rTMS may not have been sufficiently comprehensive. Third, the sample size was relatively small. Further validation of our findings in other populations of psychiatric practitioners is needed to investigate knowledge and attitudes about rTMS to generalize our findings.

## Conclusions

Chinese psychiatrists’ knowledge about rTMS needs to be strengthened to further apply rTMS in clinical practice. More widespread standardized training in rTMS will give psychiatrists more comprehensive knowledge about rTMS and result in more positive attitudes about its implementation for the treatment of refractory mental disorders.

## Supplementary information

**Additional file 1: Table S1.** Questionnaire.

## Data Availability

The datasets used and analyzed during the current study are available from the corresponding author on reasonable request.

## Abbreviations

rTMS: Repetitive transcranial magnetic stimulation
FDA: U.S. Food and Drug Administration
ORs: Odds ratios
Cis: Confidence intervals
