# Supplementary material for: Knowledge and attitudes about transcranial magnetic stimulation among psychiatrists in China
Source: BMC Psychiatry. 2020 Aug 24;20:416. doi: 10.1186/s12888-020-02817-4 (PMC7444247; doi:10.1186/s12888-020-02817-4)
Supplement: Supplementary file 1 — Additional file 1: Table S1. Questionnaire. [file 12888_2020_2817_MOESM1_ESM.docx]

| **Table S1. Questionnaire.** | | | | | | |
| --- | --- | --- | --- | --- | --- | --- |
| **No.** | **Questionnaire items** | **Possible Reponses** | | | | |
| **Sociodemographic** | |  |  |  |  |  |
| 1 | Gender | Men | Women |  |  |  |
| 2 | Age |  |  |  |  |  |
| 3 | Provinces and cities |  |  |  |  |  |
| 4 | Professional title | Resident doctor | Attending physician | Associate chief physician | Chief physician |  |
| 5 | Educational background | College | Bachelor’s degree | Master’s degree | Doctoral degree | |
| 6 | Years of work |  |  |  |  |  |
| 7 | Type of hospital | Public hospital level I | Public hospital level II | Public hospital level III |  |  |
| 8 | Psychiatric affiliation | General hospital | Specialized hospital |  |  |  |
| 9 | Does your hospital or department have an onsite clinical rTMS program? | No | Yes |  |  |  |
| 10 | Have you received training in rTMS theory? | No | Yes |  |  |  |
| 11 | Have you received training in rTMS manipulation? | No | Yes |  |  |  |
| **rTMS knowledge** | |  |  |  |  |  |
| 12 | Do you know FDA indications for rTMS for treatment-resistant depression? | No | Yes |  |  |  |
| 13 | How well do you know rTMS indications? | Not knowing or knowing a little | Know part of | Know most or all |  |  |
| 14 | How well do you know rTMS principles? | Not knowing or knowing a little | Know part of | Know most or all |  |  |
| 15 | How well do you know parameter settings of rTMS? | Not knowing or knowing a little | Know part of | Know most or all |  |  |
| 16 | How well do you know adverse reactions and contraindications of rTMS? | Not knowing or knowing a little | Know part of | Know most or all |  |  |
| **Attitudes about rTMS** | |  |  |  |  |  |
| 17 | Would you like to recommend rTMS alone for treatment of refractory mental disorders? | Strongly discourage | Don’t recommend | Neutral | Recommend | Strongly recommend |
| 18 | Would you like to recommend rTMS combined with other treatments for refractory mental disorders? | Strongly discourage | Don’t recommend | Neutral | Recommend | Strongly recommend |
| 19 | Do you think psychiatrists need to know about TMS? | Yes | No |  |  |  |
| 20 | Would you like to participate in rTMS training within hospital? | Certainly not | Probably not | Probably | Certainly |  |
| **Recommendations for rTMS** | |  |  |  |  |  |
| 21 | What are your recommendations for application of rTMS in the future? | Medical insurance coverage of rTMS | Enhance scientific research and optimize treatment plans | Formal training in rTMS theory and application among psychiatrists | Popularize rTMS among patients | Formulate treatment specifications for rTMS |
